# Metformin Can Alleviate the Symptom of Patient with Diabetic Nephropathy Through Reducing the Serum Level of Hcy and IL-33

**DOI:** 10.1515/med-2019-0071

**Published:** 2019-08-31

**Authors:** Ling Zhang, Jiansheng Niu, Xiumei Zhang, Wanxia He

**Affiliations:** 1 Department of Endocrinology, Second Hospital of Yulin City, Yulin 719000, Shaanxi Province, Yulin, China

**Keywords:** Metformin, IL-33, Hcy, Diabetic nephropathy

## Abstract

**Background:**

Interleukin-33 (IL-33) and homocysteine (Hcy) were found to be up-regulated in patients with diabetic nephropathy (DN), and the present study aimed to investigate whether metformin (MT) can influence the serum levels of IL-33 and Hcy in patients with DN.

**Methods:**

Sixty patients with type 2 diabetes mellitus (DM) were divided into DM group (albumin: Alb <20 mg/L), DN group (Alb >20mg/L), and DN+ MT treatment group, with 20 cases in each group. Patients in each group were treated with insulin for 3 months, and patients in DN+MT group was treated with insulin+MT for 3 months. The serum levels of IL-33, urinary microalbumin excretion rate (UAE), body mass index (BMI), total cholesterol (TC), high density lipoprotein cholesterol (HDL-C), creatinine (Cr), cystatin C (CysC) and Hcy were measured before and after medication. Twenty normal subjects were involved as control.

**Results:**

BMI, Hcy and TC were reduced and HDL-C was increased of patients had been treated with metformin and insulin. UAE, Cr, Ccr and CysC had no differences before and after treatment. The serum level of IL-33 significantly up-regulated in patients with DN, and MT treatment significantly decreased the serum level of IL-33 in patients with DN.

**Conclusion:**

Metformin could alleviate the symptom of patient with DN through decreasing the serum level of IL-33 and Hcy.

## 1 Introduction

Diabetes mellitus (DM) is one of the leading causes of chronic kidney disease worldwide. Based on data from the American diabetes association, in the U.S., there are about 17.5 million diabetic patients, suggesting that diabetes has become a big threaten to the health of human beings. DM can lead to the incidence of other diseases and even increase the chance of mortality worldwide [1]. Diabetic nephropathy (DN) is a common DM-related disease, and it can be observed among 30 to 40% of patients with either type 1 or type 2 diabetes [2]. At current stage, the management of DN is a painful process to the patients, not only because of the long course of the disease, but also the psychological burden and low quality of life caused to the patients [3, 4]. Therefore, it is urgent to explore more effective methods for treating DN.

The pathogenesis of DN remains largely unclear. Hcy is the intermediate production of methionine, it has been proved that the plasma level of Hcy is significantly up-regulated in patients with DN, which was mainly due to the decrease of excretion. Recently, a new member of interleukin (IL) that belongs to the IL-1 family, IL-33, is presented in cisplatin-induced nephropathy. Accumulating evidence suggested that IL-33 is one of the most important cytokines in the pathogenesis of various disorders, including autoimmune diseases, myocardial infarction, heart failure, and allergic pulmonary diseases [5, 6, 7]; however, in current literature, the data regarding the role of IL-33 in DN is limited. Thus, to confirm the specific roles of IL-33 and Hcy in DN is in a great need, which may help scientists and physician to develop novel therapies for improved therapeutic efficacy of current methods for the management of DN.

In current clinical applications, different treatment modalities have been applied to treat DN. Melatonin (MT) is one of the first-line medications the for the treatment of DN; however, the role of MT in patients with DN still requires further investigation. Hence, in the present study, we sought to determine whether MT can reduce the level of IL-33 in patients with DN and exert its therapeutic effects.

## 2 Material and methods

### 2.1 Patients

Sixty type 2 DM patients that hospitalized in Chongqing General Hospital between Jan 2016 and Feb 2017 were selected for this study. The study was approved by the Ethics Committee of Chongqing General Hospital. All of the participants signed informed consent before research. The patients were divided into DM group (Alb <20 mg/L), DN group (Alb >20mg/L), and DN+MT treatment group, with 20 cases in each group. It was the first therapy for all the patients. Patients in each group were treated with insulin, and patients in DN+ MT group was treated with insulin+MT, and the fasting blood glucose (FPG) of patients was controlled at ≤7mmol/L for a course of 3 months. Meanwhile, 20 healthy subjects who received the physical examination in our hospital during the same period were selected as the controls. The general data such as age and sex in each group showed no statistical significance (p>0.05).

### 2.2 Collection and detection of clinical specimens

Blood samples of 10 ml venous blood were collected in the morning from all patients and the healthy volunteers. After centrifugation at room temperature (1,000 rpm, 10 minutes), the supernatants were collected and then stored at −80°C. Some of the serum samples were directly sent to the clinical laboratory for the measurement of TG, TC, HDL-C, Cr, CysC, Hcy using automatic biochemistry analyzer (olympus, Japan) before and after medication; the serum levels of IL-33 (Cat no. ab223865; Abcam, USA) in each candidate was determined by using ELISA kit according to the manufacturer’s instructions; the urine of the patients was also collected at 8:00 a.m., and the levels of UAE in the urine of each patient was measured.

### 2.3 Statistical analysis

Data analysis was performed using SPSS software (Version 17.0, SPSS Inc., Chicago, Ill, USA). Data were presented as the mean ± standard deviation (SD). ANOVA was used to compare the mean value of each parameter among the groups. P <0.05 was considered statistically significant.

## 3 Results

### 3.1 Comparison of BMI, Hcy, TC, and HDL-C in different groups

Before the drug intervention, BMI, Hcy, TC and HDL-C in each group of diabetic patients shows no significant differences. After drug intervention, the BMI, Hcy, TC were significantly decreased in DN+MT group. Moreover, MT treatment also induced significant increase in the content of HDL-C in DN+MT group (Table 1-4, p<0.05).

**Table 1:** Comparison of BMI in different groups.

Group	Before drug intervention (kg/m^2^)	After drug intervention (kg/m^2^)
DM	22.9 ± 5.2	23.7 ± 2.6
DN	23.5 ± 1.7	22.5 ± 6.2
DN+MT	24.6 ± 4.4	18.7 ± 1.9*
Control	20.5 ± 2.3	21.4 ± 1.2

*p<0.05 v.s. before drug intervention.

**Table 2:** Comparison of Hcy in different groups

Group	Before drug intervention (μmol/ L)	After drug intervention (μmol/ L)
DM	12.1 ± 5.2	10.7 ± 4.8
DN	11.5 ± 3.4	11.5 ± 3.2
DN+MT	12.7 ± 5.6	9.3 ± 2.9*
Control	10.2 ± 1.1	11.4 ± 1.3

*p<0.05 v.s. before drug intervention.

**Table 3:** Comparison of TC in different groups

Group	Before drug intervention (mmol/ L)	After drug intervention (mmol/ L)
DM	4.82 ± 2.0	4.71 ± 1.5
DN	4.94 ± 1.4	4.56 ± 2.1
DN+MT	5.35 ± 1.9	4.23 ± 1.9*
Control	3.11 ± 1.1	3.24 ± 1.8

*p<0.05 v.s. before drug intervention.

**Table 4:** Comparison of HDL-C in different groups

Group	Before drug intervention (mmol/ L)	After drug intervention (mmol/ L)
DM	1.08 ± 0.13	1.11 ± 0.07
DN	0.94 ± 0.21	1.05 ± 0.13
DN+MT	1.05 ± 0.08	1.22 ± 0.24*
Control	0.99 ± 0.11	1.05 ± 0.16

*p<0.05 v.s. before drug intervention.

### 3.2 Comparison of UAE, CysC and Ccr in each group

Before the intervention, there was no significant difference between the serum level of UAE, CysC, Ccr among patients with diabetes. After drug intervention, the serum levels of UAE CysC, and Ccr in different groups had no significant changes (Table 5-7, p>0.05).

**Table 5:** Comparison of UAE in different groups

Group	Before drug intervention (μg/ min)	After drug intervention (μg/ min)
DM	12.08 ± 3.13	13.11 ± 5.64
DN	111.98 ± 6.21	108.14 ± 6.13
DN+MT	113.07 ± 7.01	112.29 ± 7.24
Control	10.42 ± 4.13	11.01 ± 3.76

**Table 6:** Comparison of Ccr in different groups

Group	Before drug intervention (ml/min)	After drug intervention (ml/min)
DM	97.36 ± 1.72	98.59 ± 1.65
DN	99.65 ± 1.33	101.25 ± 1.47
DN+MT	103.12± 1.29	95.29 ± 0.97
Control	80.14 ± 7.13	81.23 ± 6.59

**Table 7:** Comparison of CysC in different groups.

Group	Before drug intervention (mg/ L)	After drug intervention (mg/ L)
DM	1.94 ± 0.41	2.07 ± 0.48
DN	2.05 ± 0.21	1.92 ± 0.51
DN+MT	2.13 ± 0.28	1.87 ± 0.35
Control	1.13 ± 0.13	1.28 ± 0.16

### 3.3 Comparison of the serum level of IL-33 in different groups

Compared with the control group, the serum level of IL-33 was significantly increased in DM, DN and DN+MT groups. Either insulin or insulin+ MT treatment could decrease the serum level of IL-33 in different groups, and the effect of MT on the serum level of IL-33 in DN+MT group was more significant (Table 8, p<0.05).

**Table 8:** Comparison of the serum level of IL-33 in different groups.

Group	Before drug intervention (ng/L)	After drug intervention (ng/ L)
DM	107.12 ± 9.41	96.23 ± 7.51
DN	111.31 ± 8.72	91.85 ± 6.34*
DN+MT	108.74 ± 9.15	72.53 ± 9.21**
Control	41.02 ± 5.31	43.52 ± 4.83

*p<0.05, ***p<0.01v.s. before drug intervention.

## 4 Discussion

In the present study, the therapeutic effect of metformin for the treatment of DN was evaluated. We proved that metformin could alleviate the symptom of patient with DN by decreasing the serum levels of IL-33 and Hcy.

Metformin, as a first-line drug for the treatment of type 2 diabetes, will have an impact on the process of DN, and still remains doubtful. Hcy is the intermediate production of methionine during tissue metabolism substance, and it is also an endogenous pathogenic factor which has cellular and genetic cytotoxicity, which can play the role in the process of vascular damage through a variety of pathways. In patients with DN, especially with hyperinsulinemia, the elevated plasma level of Hcy is mainly due to the decrease of excretion [8]. Moreover, the levels of high external insulin can affect the activities of enzymes during the process of Hcy metabolism (such as MTHFR, MS, etc), which can also cause the increase of blood Hcy [9]. High Hcy can lead to oxidative stress and promote the repairing process of low density lipoprotein. On the other hand, it can also cause endothelial cell aging, reduce the synthesis of nitric oxide (NO), and increase secretion of vascular pseudohemophilia factor (VWF) and other cells. Hcy can also activate protein kinase C, promoting the expression of proto oncogene c-fos and c-myb in vascular smooth muscle cells [10, 11, 12]. Therefore, Hcy may be induced by oxidative stress and endothelial damage. The results of this study showed that Hcy level significantly enhanced in patients with type 2 DM compared with the control group, and MT treatment induced significant decrease in the expression of Hcy in patients with DN, and also altered the expression of Hcy-related molecules TC and HDL-C. Taken together, these results suggested that Hcy was involved in the process of MT-induced therapeutic effects.

IL-33 is possible to promote cell proliferation and inhibit apoptosis via activation of various pathways such as STAT3, MAPK, and/or NF-κB signaling in different types of cells [13]. Autocrine production of IL-33 has been suggested to contribute to human cancer cell survival, migration and resistance to chemotherapy, through the up-regulation of anti-apoptotic proteins including Bcl-2, and via ST2/STAT3 [14, 15]. In the field of DN-related studies, reports on the functions of IL-33 in the pathogenesis of the disease is rare. In the present study, we focused on the role of IL-33 in the management of DN using MT. It has been observed that compared with the control group, the serum level of IL-33 was significantly increased in DM and DN groups. Either insulin or insulin+ MT treatment could decrease the serum level of IL-33 in DN patients, and the effect of MT on the serum level of IL-33 in DN+MT group was more significant.

In conclusion, our results indicated that metformin could alleviate the symptom of patient with DN through reducing the serum level of IL-33. Our data has provided novel evidence for the application of IL-33 as an index for evaluating the therapeutic efficacy of MT for the treatment of DN.
